# Supplementary material for: Causal Relationship between Aging and Anorexia Nervosa: A White-Matter-Microstructure-Mediated Mendelian Randomization Analysis
Source: Biomedicines. 2024 Aug 16;12(8):1874. doi: 10.3390/biomedicines12081874 (PMC11351342; doi:10.3390/biomedicines12081874)
Supplement: Supplementary file 1 [file biomedicines-12-01874-s001.zip › Supplementary Figure/Figure S4/Singe SNP plot of MR analysis for TL to ubm-b-1569.pdf]

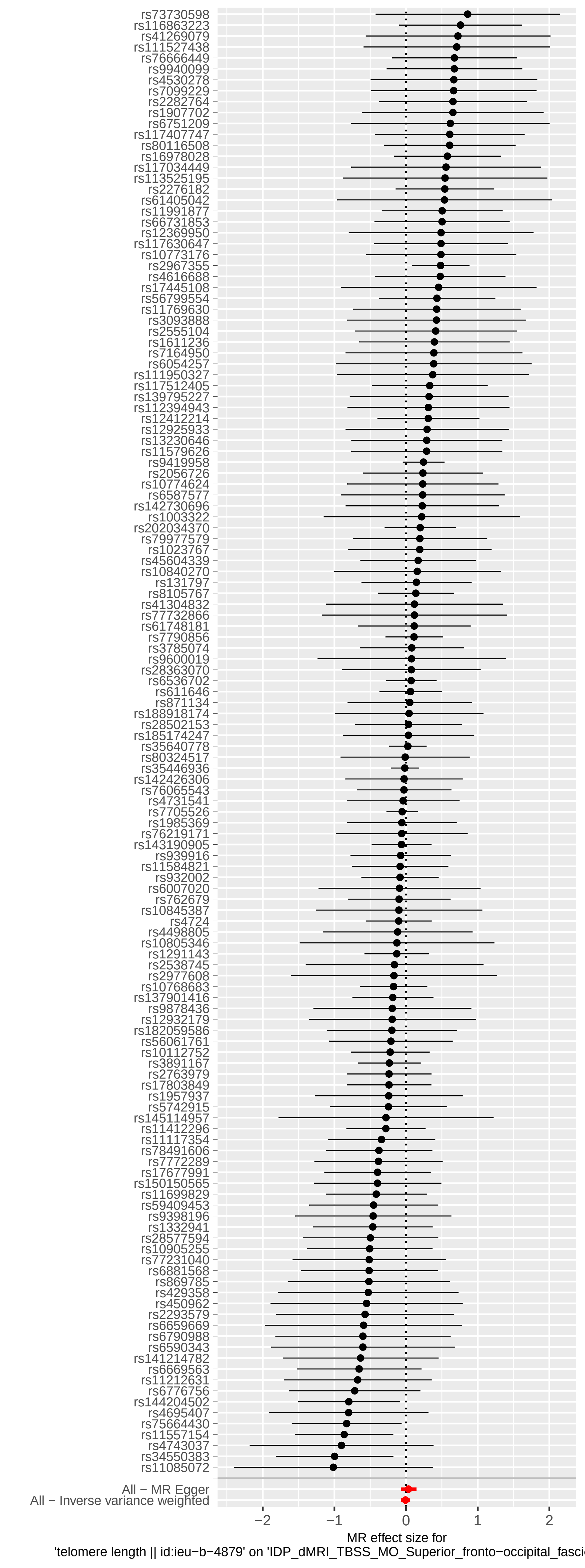
rs73730598
rs116863223
rs41269079
rs111527438
rs76666449
rs9940099
rs4530278
rs7099229
rs2282764
rs1907702
rs6751209
rs117407747
rs80116508
rs16978028
rs117034449
rs113525195
rs2276182
rs61405042
rs11991877
rs66731853
rs12369950
rs117630647
rs10773176
rs2967355
rs4616688
rs17445108
rs56799554
rs11769630
rs3093888
rs2555104
rs1611236
rs7164950
rs6054257
rs111950327
rs117512405
rs139795227
rs112394943
rs12412214
rs12925933
rs13230646
rs11579626
rs9419958
rs2056726
rs10774624
rs6587577
rs142730696
rs1003322
rs202034370
rs79977579
rs1023767
rs45604339
rs10840270
rs131797
rs8105767
rs41304832
rs77732866
rs61748181
rs7790856
rs3785074
rs9600019
rs28363070
rs6536702
rs611646
rs871134
rs188918174
rs28502153
rs185174247
rs35640778
rs80324517
rs35446936
rs142426306
rs76065543
rs4731541
rs7705526
rs1985369
rs76219171
rs143190905
rs939916
rs11584821
rs932002
rs6007020
rs762679
rs10845387
rs4724
rs4498805
rs10805346
rs1291143
rs2538745
rs2977608
rs10768683
rs137901416
rs9878436
rs12932179
rs182059586
rs56061761
rs10112752
rs3891167
rs2763979
rs17803849
rs1957937
rs5742915
rs145114957
rs11412296
rs11117354
rs78491606
rs7772289
rs17677991
rs150150565
rs11699829
rs59409453
rs9398196
rs1332941
rs28577594
rs10905255
rs77231040
rs6881568
rs869785
rs429358
rs450962
rs2293579
rs6659669
rs6790988
rs6590343
rs141214782
rs6669563
rs11212631
rs6776756
rs144204502
rs4695407
rs75664430
rs11557154
rs4743037
rs34550383
rs11085072
All − MR Egger
All − Inverse variance weighted
−2
−1
0
1
2
MR effect size for
'telomere length || id:ieu−b−4879' on 'IDP_dMRI_TBSS_MO_Superior_fronto−occipital_fasci
